# Spectrum of *DICER1* Germline Pathogenic Variants in Ovarian Sertoli–Leydig Cell Tumor

**DOI:** 10.3390/jcm10091845

**Published:** 2021-04-23

**Authors:** Elisa De Paolis, Rosa Maria Paragliola, Paola Concolino

**Affiliations:** 1 Molecular and Genomic Diagnostics Unit, Fondazione Policlinico Universitario A. Gemelli IRCCS, Largo A. Gemelli 8, 00168 Rome, Italy; paola.concolino@policlinicogemelli.it; 2 Department of Translational Medicine and Surgery, Università Cattolica del Sacro Cuore, Largo F. Vito 1, 00168 Rome, Italy; rosamaria.paragliola@policlinicogemelli.it; 3 Unit of Endocrinology, Fondazione Policlinico Universitario A. Gemelli IRCCS, Largo A. Gemelli 8, 00168 Rome, Italy

**Keywords:** Sertoli–Leydig Cell Tumors, DICER1, molecular diagnosis

## Abstract

Sertoli–Leydig Cell Tumors (SLCTs) are rare ovarian sex cord-stromal neoplasms, which predominantly affect adolescents and young female adults. The SLCTs clinical diagnosis and treatment remains challenging due to the rarity and the varied presentation. A large majority of SLCTs are unilateral, but also bilateral neoplasms have been reported, sometimes in the context of *DICER1* syndrome. In fact, the most significant discovery regarding the molecular genetics basis of SLCTs was the finding of somatic and germline pathogenic variants in the *DICER1* gene. The DICER1 protein is a key component of the micro-RNA processing pathway. Germline *DICER1* pathogenic variants are typically inherited in an autosomal dominant pattern and are most often loss-of-function variants dispersed along the length of the gene. Contrarily, *DICER1*-related tumors harbor a characteristic missense “RNase IIIb hotspot” mutation occurring *in trans*, or, less frequently, loss of heterozygosity (LOH) event involving the wild-type allele. While *DICER1* mutations have been identified in approximately 60% of SLCTs, especially in the moderately or poorly differentiated types, there are only a few case reports of ovarian SLCT with underlying germline *DICER1* mutations. In this review, we focus on the molecular genetic features of SLCT, performing an extensive survey of all germline pathogenic variants modifying the whole sequence of the *DICER1* gene. We point out that *DICER1* genetic testing, coupled with an accurate variants classification and timely counseling, is of crucial importance in the clinical management of ovarian SLCT-affected patients.

## 1. Introduction

Ovarian Sex Cord-Stromal Tumors (SCSTs) represent approximately 8% of ovarian cancers and comprise a heterogeneous group of malignancies. These tumors are formed by different cell types that arise from the primitive sex cords or stromal cells. Granulosa cell tumors (GCTs) represent 90% of malignant SCSTs, whereas Sertoli–Leydig Cell Tumor (SLCT) account for less than 0.5% of all ovarian cancers and for ≈15% of all ovarian stromal cell tumors [1,2]. The low incidence of SLCTs reflects in part the insufficiency of data regarding the clinical behavior of these tumors and their oncological outcomes. SLCTs are usually predominant in the 2nd and 3rd decades of life and they are characterized by the presence of a testicular structure that produces androgens. Signs and symptoms of virilization appear in two phases, characterized by defeminization followed by the phase of virilization [3]. At premenopausal age, women experience oligomenorrhea or amenorrhea, while virilization can appear with hirsutism, acne, frontal hair thinning, male pattern baldness, deepening of the voice, increased musculature, increased libido, and clitoromegaly [4,5,6,7]. SLCTs can also present with abdominal pain and increased abdominal circumference, frequently with a palpable adrenal mass at physical examination [8]. From a prognostic evaluation of patients, an important feature is the SLCTs degree of differentiation. According to the classification of Female Genital Tumors proposed by World Health Organization (WHO), SLCTs can be subdivided into well-differentiated, moderately differentiated, and poorly differentiated based on the degree of differentiation of the sertoliform component [9]. Well-differentiated tumors show a practically null malignant potential, whereas the risk increases substantially in those with lower degrees of differentiation [10,11]. Most of the ovarian SLCTs are unilateral and diagnosed in stage I, and their treatment is surgical [2,6,11,12]. Moreover, unilateral salpingo-oophorectomy is the appropriate treatment in patients that want to preserve their fertility [5,6,10]. In older patients, as well as in advanced stages, total hysterectomy and bilateral salpingo-oophorectomy or cytoreductive surgery are indicated [4,13]. Lymph node metastasis in ovarian SCSTs is rare, but relapse is not uncommon especially in advanced-stage disease (stage II to IV) [14,15].

Data from Karnezis et al. suggest at least three molecular subtypes of SLCT with distinct clinicopathologic features: *DICER1* mutant (younger, more androgenic symptoms, moderately/poorly differentiated, retiform, or heterologous elements), *FOXL2* mutant (postmenopausal, abnormal bleeding, moderately/poorly differentiated, no retiform or heterologous elements), and *DICER1/FOXL2* wild type (intermediate age, no retiform or heterologous elements, including all well-differentiated tumors) [16]. The *DICER1*-mutant type is the most common type. In fact, the most significant discovery regarding the molecular genetics of SLCT was the finding of underlying somatic and germline mutations in the *DICER1* gene, with the occurrence of SLCT as part of an autosomal dominant disorder named *DICER1* syndrome [17,18,19]. *DICER1* syndrome, also known as pleuropulmonary blastoma family tumor susceptibility syndrome, shows a large spectrum of clinical phenotypes. Affected individuals are at increased risk of developing pleuropulmonary blastoma, cystic nephroma, rhabdomyosarcoma, multinodular goiter, thyroid cancer, ovarian SLCTs, and other neoplasia. The majority of tumors occur in individuals younger than 40 years [17,18,19,20,21]. As mentioned above, *DICER1* variants are typically inherited in an autosomal dominant pattern, but they may also arise de novo in the germline or in a somatic mosaic distribution [21].

Here, we present a literature review of all *DICER1* germline pathogenic variants reported in patients with ovarian SLCTs. A spectrum of *DICER1* germline alterations was provided, together with their comprehensive molecular and clinical description.

## 2. Genetics of *DICER1*

The human *DICER1* gene is located on chromosome 14q32.13 and consists of 27 exons encoding for a 1922 amino-acid long protein (200 kDa) [21]. DICER1 is an endoribonuclease protein belonging to the ribonuclease III family and representing a key element of the RNA interference pathway. In particular, DICER1 is a component of the RNA-induced silencing complex (RISC) loading complex (RLC), which is composed of DICER1, Argonaute-2 (AGO2), and trans-activation-responsive RNA binding protein 2 (TARBP2). The DICER1 protein domains include Helicase 1/2, ATP-binding domain, Helicase, C-terminal domain, Dicer dimerization domain (DDD), PAZ domain (PAZ), ribonuclease IIIa domain, and ribonuclease IIIb domain [17]. DICER1 cleaves precursor-microRNA molecules (pre-miRNA) in the citoplasm, producing ≈20–22 nucleotide-long mature regulatory microRNAs (miRNA) [22]. Then, each miRNA is linked to the RISC. The newly formed RISC–miRNA complex binds to specific mRNA, inhibiting ribosomal access and the subsequent translation [23]. Dysregulation of miRNA production is related to a pro-oncogenic effect, as observed in several tumor types. In fact, the overexpression of one miRNA may inhibit the protein translation of a cancer suppressor gene, while the downregulation of another miRNA may increase the protein level of an oncogene [24,25,26].

Some aspects of *DICER1* genetics are unique and need to be pointed out. The prevalence of germline *DICER1* pathogenic variants has been estimated to be 1/10,600 in the general population [27]. These mutations are most often loss-of-function (LoF) variants dispersed along the length of the protein and are typically inherited in an autosomal dominant pattern, showing an incomplete penetrance [20,28]. The predicted functional effect of all the germline *DICER1* LoF variants is essentially equivalent, resulting in a complete or near-complete LoF in miRNA processing. This prediction is based partly on nonsense-mediated decay, but it also reflects the functional domain structure of the *DICER1* protein [29]. In addition, the presumed equivalence of *DICER1* LoF mutations is also consistent with clinical findings: no correlations are reported between the locations of germline LoF mutations within the *DICER1* gene and clinical features, such as age of disease onset, number of disease foci, specific tissue sites involved, or survival [29]. Moreover, *DICER1* is not a classical tumor suppressor gene for which “two hits”—loss of function in both alleles—are required to allow tumorigenesis. In fact, in the *DICER1* syndrome, the related tumors typically acquire a second missense somatic mutation (second hit) within the RNase IIIb domain that does not fully take out *DICER1* ability but causes neomorphic DICER1 function in miRNA processing: the cleavage of mature 5p miRNAs from the 5′ end of pre-miRNA hairpin structures fails, while mature 3p miRNAs continue to be cleaved from the 3′ end normally [29]. This neomorphic function leads to the tumor suppression to falter when the expression of wild-type *DICER1* allele is impaired due to an LoF variant or a loss of heterozygosis (LOH) event [28,29]. So, the two mutational events, the RNase IIIb missense and the LoF alterations, are required to promote the initiation of tumorigenesis. However, the occurring of a somatic mutation specifically affecting the RNase IIIb domain is considered a low-probability event, while a LoF mutation along the gene is, relatively, a high-probability event. The consequence of these lopsided probabilities is that the occurrence of an RNase IIIb hotspot mutation becomes the rate-limiting step in the onset of pathogenesis [20,29]. In addition, also the timing in which the second hit occurs is crucial. As proposed, there are apparently windows of risk for neoplastic transformation, coinciding, for example, with specific periods of organ/tissue development. This further lowers the odds of all events coinciding, and conceptually, it could explain the reduced penetrance and the variable expression of familial LoF mutations in *DICER1* syndrome [29,30]. In fact, the penetrance of *DICER1* pathogenic variants is generally low and is higher in female than in males. Stewart et al. reported that ≈5% of non-proband *DICER1* heterozygotes were documented to have developed a neoplasm by age 10 years, which increased to ≈20% by age 50 years [30].

While most individuals with *DICER1* syndrome are heterozygous for a germline LoF *DICER1* pathogenic variant, other predisposing *DICER1* alterations have also been documented as somatic mosaicism [31,32]. In particular, individuals with mosaicism for RNase IIIb hotspot mutations demonstrate an increased penetrance of *DICER1* syndrome including earlier onset, greater number of disease foci, and greater range of phenotypes. In these cases, the first hit is the acquisition of the missense RNase IIIb hotspot mutation, while the second loss-of-function mutation can then occur anywhere across the coding region and therefore is stochastically more probable [20,29].

Finally, it was hypothesized that a *DICER1* hotspot missense mutation can be sufficient to promote tumorigenesis, even in the presence of an expressed wild-type allele [32,33]. A dominant-negative effect of a RNase IIIb hotspot mutant protein over the wild-type DICER1 could account during the miRNA processing, as demonstrated by Rakheja et al. for DROSHA protein [33].

## 3. *DICER1* Somatic Mutations in Ovarian Sertoli–Leydig Cell Tumor

In the recent years, many studies have investigated the molecular basis of ovarian SCST [34,35,36,37,38,39,40,41,42]. In 2012, using a combination of whole transcriptome and whole exome sequencing analyses, Heravi-Moussavi et al. sequenced 14 non-epithelial ovarian tumors identifying closely clustered missense mutations in the region of *DICER1* gene encoding the RNase IIIb domain in a total of four samples [35]. Successively, the authors explored this region by Sanger sequencing, in additional ovarian tumors samples discovering *DICER1* mutations in 30 of 102 non-epithelial ovarian tumors (29%). In particular, DICER1 mutations have been identified in approximately 60% of SLCTs (26 of a total of 43) [35]. These mutations were restricted to codons encoding metal-binding sites (p.Glu1705, p.Asp1709, p.Asp1810, p.Glu1813) within the RNase IIIb catalytic centers, which are critical for microRNA interaction and cleavage. Among these, the p.Asp1709Asn (c.5125G>A) was the most common somatic mutation identified in ovarian SLCTs samples (10/26, 38%) [35]. To date, the reason for this clusterization is not fully understood. It was proposed that these mutations specifically affect the processing of miRNA [33,43], potentially conferring a selective growth advantage and thus, they may be selected for by a tumor cell [20].

A subsequent study performed by Conlon et al. confirmed that *DICER1* hotspot mutations occur in over half of ovarian SLCTs. The authors subjected 32 ovarian SLCTs to *DICER1* hotspot mutation analysis using Sanger sequencing. Twenty of 32 (63%) tumors harbored a *DICER1* hotspot mutation, of which 80% had the p.Glu1705Lys variant [40]. In 2017, de Kock et al. reported a very high frequency of *DICER1* mutations in SLCTs: of 34 cases diagnosed as SLCTs, a total of 30 (88%) harbored ≥1 *DICER1* mutations [42]. Interestingly, all the 30 moderately differentiated or poorly differentiated SLCTs harbored *DICER1* mutations, while all four well-differentiated SLCTs were *DICER1* wild type [41]. Based on these results, the authors speculated that well-differentiated SLCTs constitute a unique entity with a different pathogenesis from moderately differentiated and poorly differentiated SLCTs [42]. These observations have been confirmed by subsequent studies: all of the SLCTs with *DICER1* mutations reported to date have been moderately and poorly differentiated; none of the well-differentiated tumors examined to date have been shown to harbor *DICER1* pathogenic variants [16].

## 4. DICER1 Pathogenic Germline Variants in Ovarian Sertoli–Leydig Cell Tumor

### 4.1. Literature Review

We have catalogued all *DICER1* germline pathogenic variants reported in articles published before February 28, 2012. We choose to consider only papers in which patient’s clinical information was provided. Consequently, we decide to exclude all those variants reported in common public databases (e.g., Exome Aggregation Consortium (ExAC), Genome Aggregation Database (gnomAD), and 1000 Genomes) that are missing that kind of data. Articles were identified via National Center for Biotechnology Information (NCBI) PubMed literature database using the search terms “*DICER1* germline mutations and ovarian Sertoli–Leydig Cell Tumor” or “*DICER1* germline variants and ovarian Sertoli–Leydig Cell Tumor”. More than 100 papers were read and evaluated for the reliable presence of *DICER1* germline variants. The reference list was double checked for additional publications. After the literature review, 32 articles were identified to contain relevant information. We decided to report the identified variants according to Locus Reference Genomic (LRG) *DICER1* reference sequence NM_177438.2. Table 1 provides the list of all the 43 *DICER1* pathogenic variants related to ovarian SLCT. A full description of each variant was showed, including nucleotide change, amino acid change, *DICER1* exon/intron number, and the protein domain involved in the mutation. The germline variant classification was assessed using VarSome tool (https://varsome.com, V6.7, Lausanne, Switzerland; last access date 23 April 2021). If a concomitant *DICER1* somatic mutation was described in the same patient, it was reported in brackets below the germinal variant, as for its relative information. In addition, we provided a description of the clinical phenotype of the *DICER1*-mutated patient (to notice, when more individuals belonging to the same family were affected by SLCT, only the proband was considered; moreover, probands belonging to different families and carrying the same variants were individually described in Table 1). In the reference column, the paper identifying the clinical case was reported.

### 4.2. The Patients

A total of 57 independent cases were identified. The age at diagnosis of the SLCT was known for 46 patients with a median of 17.6 years. Even if the histological information of some SLCTs reviewed was missed in the corresponding reference papers, it is reasonable to assume that these tumors were all poorly or moderately differentiated. Nine cases of bilateral SLCT were reported. In addition to SLCT, the majority of the patients presented other DICER1 syndrome-associated diseases. In particular, an increased incidence of thyroid lesions was documented: 19 patients with Multinodular Goiter (MNG), 3 with Papillary Thyroid Carcinoma (PTC), 4 with Differentiated Thyroid Carcinoma (DTC), 4 with a thyroid nodule, 1 affected by Thyroid Follicular Adenoma (TFA), and 1 presented a thyroid cyst (Table 1). Based on these findings, some authors suggest that the association between thyroid nodules/thyroid carcinoma and SLCT is highly suggestive for DICER1 syndrome. Interestingly, in the majority of the reported cases, the diagnosis of SLCT has been made on the basis of abdominal pain and/or abdominal/pelvic mass, while data about clinical and/or biochemical hyperandrogenism are available only in few cases. In the study of Yuan et al., eleven patients had androgenic manifestations, three patients presented symptoms related to an inappropriate estrogen secretion, while three patients did not have any manifestation associated with elevated secretion of sex hormones [46]. The patient reported by Luke et al. represents the first case of secondary amenorrhea due to elevated inhibin B levels in a female adolescent with an ovarian SLCT [55]. The two siblings with *DICER1* syndrome described by Zhang et al. were diagnosed with ovarian mass and irregular menstruation, but serum testosterone levels were normal in both cases [57]. Elevated testosterone levels associated with signs of hyperandrogenism have been reported in 3 cases [60,62,67].

### 4.3. Germline DICER1 Alterations

**Figure 1:**
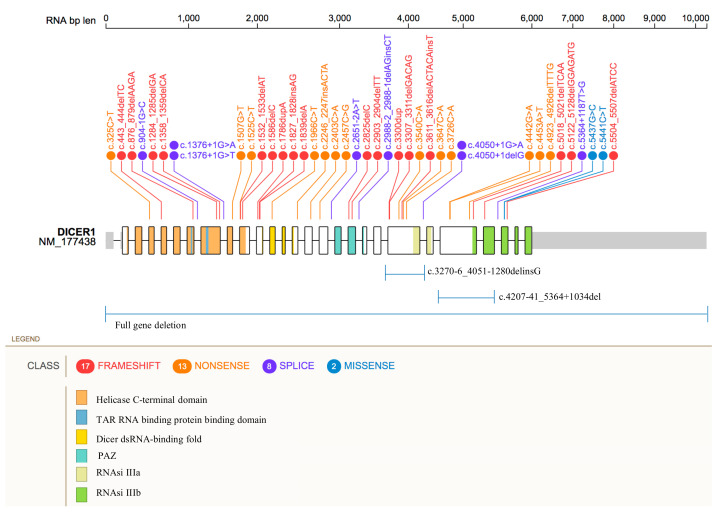
Distribution of *DICER1* germline pathogenic variants. Figure 1 shows the location of the genetic variants along the *DICER1* gene. SNPs and *indels* mutations are reported as frameshift (red), nonsense (orange), splice-site (purple), and missense (blue) variants. Large deletions are shown below. Protein domains are represented by colored areas.

Unlike somatic mutations, which are preferentially located within the RNase IIIb domain at specific hotspot residues, as previously described, pathogenic and likely pathogenic germline variants identified in SLCTs are found to be scattered along the full length of the *DICER1* gene (Figure 1).

**Figure 2:**
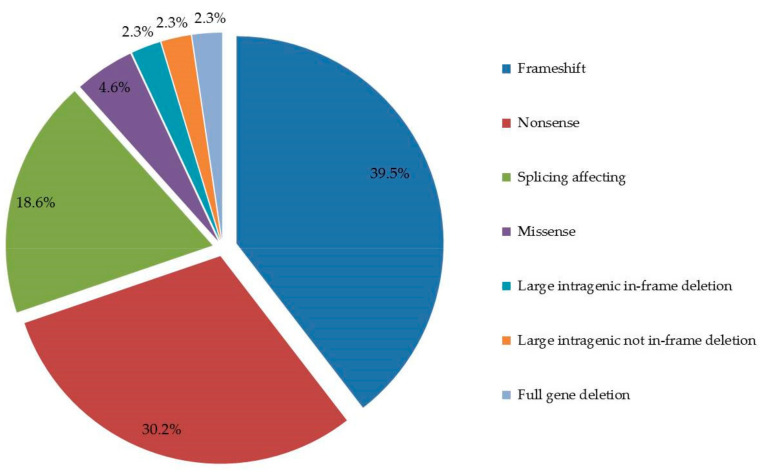
*DICER1* pathogenic variants types. The figure shows the pie chart representative of the numerical proportion of the *DICER1* genetic variants types.

According to our results, the 43 *DICER1* germline variants consist of 39.5% (17/43) frameshift mutations, 30.2% (13/43) nonsense mutations, 18.6% (8/43) splicing affecting variants, 4.6% (2/43) missense variants 2.3% (1/43), large intragenic in-frame deletion, 2.3% (1/43) large intragenic not in-frame deletion and, finally, 2.3% (1/43) full gene deletion (Figure 2).

Taking into account the distribution of the pathogenic single nucleotide variants (SNVs) and indels accounted in *DICER1* gene, the exons carrying the largest number of different mutations were the numbers 21 and 23, harboring a total of six and four pathogenic variants, respectively. To note, these exons are involved also in the two intragenic large deletions, as reported in Table 1. In the remaining exons, only a few mutations (from one to three) were identified, and no pathogenic variants associated to ovarian SLCT were reported within the exons 1–3, 6, 13, 17, 19, 20, and 22. In addition, no different variants, affecting the same codon, have been identified, whereas the donor-splicing sites of introns 8 and 21 were both affected by two different mutations at +1G position (Table 1, Figure 1). The Helicase C-terminal domain, the region between PAZ and RNase IIIa and the RNase IIIb domains resulted as the DICER1 domains harboring the highest number of mutations, with a total of five, seven, and six, respectively.

To note, the two missense variants reported in Table 1 (c.5437G>C; c.5441C>T) are classified as pathogenic variants taking also into account the Varsome bioinformatic tool verdict. In fact, multiple computational evidences support a deleterious effect on the gene product. In addition, the above-mentioned variants are not reported in controls from the Exome Sequencing Project, 1000 Genomes Project, or Exome Aggregation Consortium.

Among the 43 identified variants, only the c.5437G>C (p.Glu1813Gln) was reported as germline mosaicism [31]. In the corresponding clinical case, the alternative C allele was detected in saliva-derived DNA with a frequency of 0.25%, but it was not found in the patient’s blood DNA. In addition, a second deleterious somatic variant, the c.4626delG (p.Gln1542HisfsTer18), was identified in patient’s left ovarian SLCT, whereas the *DICER1* loss of heterozygosity (LOH) was detected in the right ovarian SLCT [31].

Finally, here, we mention the *DICER1* c.5096-12G>A variant. This germline intronic substitution was found by Schultz et al. in two adolescents with SLCT [72]. It results in an exact duplication of six bases at the splice site of the intron 23 and exon 24 junction [72]. The predicted improper splicing would lead to the inclusion in the coding sequence of 10 bases of the intronic sequence, with the consequent frameshift and premature truncation of the protein (p.Asp1699AlafsTer8) that disrupts the RNase IIIb domain. To date, the ClinVar database (https://www.ncbi.nlm.nih.gov/clinvar/. Last access date 23 April 2021) and the VarSome tool classify the c.5096-12G>A substitution as Variant of Uncertain Significance (VUS). In fact, the predicted splicing error has not been confirmed by published transcriptional studies. In conclusion, the currently available evidences are considered insufficient to determine the exact role of this variant in the disease.

## 5. Discussion

In recent years, many studies have investigated the molecular basis of ovarian SLCT. Consistent literature data support the role of DICER1 in the disease pathogenesis [13,14,34,35,36,37,38,39,40,41,42]. In this article, we present a comprehensive update of the germline *DICER1* alterations identified in SLCTs over the past 10 years. A total of 43 pathogenic variants were collected (Table 1). These have been recognized as nonsense or frameshift mutations, leading to stop codons and truncated proteins, or nonsense-mediated RNA degradation. No mutational germline hotspots were identified.

Although rare, *DICER1* syndrome can be associated with significant morbidity, often occurring at a young age. For this reason, it is important to emphasize the clinical relevance of *DICER1* germline genetic testing, with the aim of detecting precancerous or lower-stage lesions [64,73]. In particular, the prompt diagnosis of SLCTs at an early stage could help avoid chemotherapy and improve survival [74]. Moreover, as recently reported by Yuan et al., the information regarding *DICER1* molecular testing could have a prognostic value in SLCT clinical management, where patients harboring germline alterations may be more likely to exhibit a clinical relapse [46]. Therefore, in this context, genetic counseling, molecular sequencing analysis, and variant classification assume a decisive importance.

Full gene Sanger sequencing was the most used approach for the identification of germline *DICER1* variants reported in Table 1. However, this approach may fail to detect somatic variants and mosaicism events, if accounted as low frequency alterations. On the contrary, high-throughput techniques such as Next-Generation Sequencing (NGS) performed on blood, tumor tissues, and also non-tumor samples improve the diagnostic confidence of the molecular test [29,46,49,57,59]. In our opinion, an NGS approach could also allow the prediction of Copy Number Variations (CNVs) via bioinformatics analysis of row sequencing data. In this case, the Multiplex Ligation Probe Amplification (MLPA) test could be used as a confirmatory method [58]. Finally, an integrate workflow encompassing NGS, Sanger sequencing, and MLPA is recommended [63].

We want to point out the need of the spread of *DICER1* molecular testing in suggestive patients’ management due to the above-mentioned advantages and considering that this is, to date, an uncommon diagnostic practice. During our literature review analysis, we found several SLCT case reports in which the genetic test was not performed. We can speculate that the main reason is to be found above all in the difficulty in testing *DICER1* gene status. In this context, the present review intended to be a support for clinicians in terms of diagnosis, management, and familiar counseling of SLCT-affected women, but also in the more comprehensive context of *DICER1*-syndrome affected patients.

## Figures and Tables

**Table 1:** Spectrum of *DICER1* pathogenic variants reported in ovarian SLCT. If described in the corresponding case report, the concomitant somatic mutation is reported in bracket below the germline one.

Coding DNA Reference Sequence ^&^	Exon/Intron	Codon	Protein Change	Protein Domain ^Δ^	Variant Classification ^◊^	Patient Phenotype ^†^	Ref.
c.325C>T	4	109	p.Gln109Ter	Helicase ATP-binding	Pathogenic	SLCT (7y), MNG (11y)	[44]
c.325C>T	4	109	p.Gln109Ter	Helicase ATP-binding	Pathogenic	MNG, SLCT (38y)	[45]
c.443_444delTC* (c.5425G>A)	5 (25)	148 (1809)	p.Leu148HisfsTer22 (p.Gly1809Arg)	Helicase ATP-binding (RNase IIIb)	Pathogenic	SCLT (14y)	[46]
c.876_879delAAGA	7	293	p.Arg293Ile*fs*Ter4	TAR RNA binding protein binding domain	Pathogenic	MNG (9y), SLCT (14y), Polyps-GI	[47]
c.904-1G>C (c.5439G>A)	Intron 7 (25)	- (1813)	- (p.Glu1813Glu)	-	Likely Pathogenic	MNG (12y), cERMS (17y), SLCT (18y)	[48]
c.1284_1285delGA (c.5437G>C)	8 (25)	429 (1813)	p.Lys429AlafsTer47 (p.Glu1813Gln)	TAR RNA binding protein binding domain (RNase IIIb)	Pathogenic	SLCT (54y), Pulmonary Bullae	[42]
c.1358_1359delCA (c.5125G>A)	8 (24)	453 (1709)	p.Thr453SerfsTer23 (p.Asp1709Asn)	Helicase C-terminal (RNase IIIb)	Pathogenic	SLCT (12y)	[46]
c.1376+1G>T	Intron 8	-	-	-	Pathogenic	SLCT	[34]
c.1376+1G>T	Intron 8	-	-	-	Pathogenic	PPB type II, MNG, Thyroid-PTC, SLCT	[49]
c.1376+1G>T	Intron 8	-	-	-	Pathogenic	PPB Type II, NCMH, SLCT, Thyroid-DTC, Peritoneal Cyst	[29]
c.1376+1G>T	Intron 8	-	-	-	Pathogenic	PPB (70y), NCMH (13y), Thyroid-PTC, SLCT	[50]
c.1376+1G>A (c.5439G>T)	Intron 8 (25)	- (1813)	- (p.Glu1813Asp)	- (RNase IIIb)	Pathogenic	PPB-Type-II (5.5y),Thyroid-DTC (8y), SLCT (13.5y), NCMH (13.5y)	[51]
c.1376+1G>A (c.5439G>T)	Intron 8 (25)	- (1813)	- (p.Glu1813Asp)	- (RNase IIIb)	Pathogenic	SLCT (13y), PPB, Thyroid-Nodule, Nasal Polyp/s	[42]
c.1507G>T	9	503	p.Glu503Ter	Helicase C-terminal	Pathogenic	SLCT	[34]
c.1525C>T	10	509	p.Arg509Ter	Helicase C-terminal	Pathogenic	SLCT-bilateral (8y, 14y), MNG (14y)	[52]
c.1525C>T (c.5125G>A)	10 (24)	509 (1709)	p.Arg509Ter (p.Asp1709Asn)	Helicase C-terminal (RNase IIIb)	Pathogenic	SLCT (13y), Thyroid-Nodule	[42]
c.1532_1533delAT (c.5429A>T)	10 (25)	511 (1810)	p.His511ArgfsTer16 (p.Asp1810Val)	Helicase C-terminal (RNase IIIb)	Pathogenic	SLCT (16y)	[53]
c.1532_1533delAT (c.5438A>C)	10 (25)	511 (1813)	p.His511ArgfsTer16 (p.Glu1813Ala)	Helicase C-terminal (RNase IIIb)	Pathogenic	SLCT (28y), Thyroid-Cyst	[42]
c.1532_1533delAT (c.5113G>A)	10 (24)	511 (1705)	p.His511ArgfsTer16 (p.Glu1705Lys)	Helicase C-terminal (RNase IIIb)	Pathogenic	SLCT (15y)	[42]
c.1532_1533delAT (c.5429A>T)	10 (25)	511 (1810)	p.His511ArgfsTer16 (p.Asp1810Val)	Helicase C-terminal (RNase IIIb)	Pathogenic	SLCT (16y)/JGCT	[42]
c.1586delC (c.5437G>C)	10 (25)	529 (1813)	p.Pro529GlnfsTer33 (p.Glu1813Gln)	Helicase C-terminal (RNase IIIb)	Pathogenic	SLCT-bilateral (20y, 29y)	[54]
c.1786dupA (No somatic variant detected)	11	596	p.Thr596AsnfsTer3	Helicase C-terminal	Pathogenic	SLCT (59y)	[46]
c.1827_1828insAG	11	610	p.Val610Arg*fs*Ter8	Between Helicase C-terminal and Dicer dsRNA-binding fold	Pathogenic	PPB Type Ir, CN, SLCT	[29]
c.1839delA	11	614	p.Tyr614Met*fs*Ter3	Between Helicase C-terminal and Dicer dsRNA-binding fold	Pathogenic	SLCT-bilateral (8y, 14y)	[55]
c.1966C>T	12	656	p.Arg656Ter	Dicer dsRNA-binding fold	Pathogenic	SLCT-bilateral (12y,14y)	[56]
c.2246_2247insACTA	14	749	p.Tyr749Ter	Between Dicer dsRNA-binding fold and PAZ	Pathogenic	SLCT	[34]
c.2403C>A (c.5113G>A)	15 (24)	801 (1705)	p. Cys801Ter (p.Glu1705Lys)	Between Dicer dsRNA-binding fold and PAZ (RNase IIIb)	Pathogenic	MNG (20y), SCLT (21y)	[57]
c.2457C>G	16	819	p. Tyr819Ter	Between Dicer dsRNA-binding fold and PAZ	Pathogenic/Likely Pathogenic	MNG (9y), SLCT (14y)	[47]
c.2457C>G (c.5437G>A)	16 (25)	819 (1813)	p.Tyr819Ter (p.Glu1813Lys)	Between Dicer dsRNA-binding fold and PAZ (RNase IIIb)	Pathogenic/Likely Pathogenic	MNG (9y), SLCT (14y)	[35]
c.2651-2A>T* (c.5425G > A)	Intron 16 (25)	- (1809)	- (p.Gly1809Arg)	- (RNase IIIb)	Pathogenic	SLCT (14y)	[46]
c.2825delC (c.5125G>A)	18 (24)	942 (1709)	p.Pro942LysfsTer6 (p.Asp1709Asn)	PAZ (RNase IIIb)	Pathogenic	SLCT	[35]
c.2903_2904delTT (c.5437G>C)	18 (25)	968 (1813)	p.Phe968CysfsTer5 (p.Glu1813Gln)	PAZ (RNase IIIb)	Pathogenic	SLCT (20y)	[42]
c.2988-2_2988-1delAGinsCT	Intron 18	-	-	-	Pathogenic	WT (6y), MNG (9y), SLCT (12y)	[56]
c.3270-6_4051-1280delinsG	21	1091	p.Tyr1091Ser*fs*Ter28	Between PAZ and RNase IIIa	Pathogenic	SLCT (6y), MNG (14y)	[58]
c.3300dup (c.5437G>A)	21 (25)	1101 (1813)	p.Ser1101IlefsTer3 (p.Glu1813Lys)	Between PAZ and RNase IIIa (RNase IIIb)	Pathogenic	SLCT (53y)	[42]
c.3300dup (c.5113G>A)	21 (24)	1101 (1705)	p. Ser1101IlefsTer3 (p.Glu1705Lys)	Between PAZ and RNase IIIa (RNase IIIb)	Pathogenic	PPB-Type-I (1y), LC (1.8y), SLCT (18y)	[42]
c.3307_3311delGACAG (c.5439G>T; c.5439G>C)	21 (25)	1103 (1813)	p.Asp1103GlnfsTer8 (p.Glu1813Asp)	Between PAZ and RNase IIIa (RNase IIIb)	Pathogenic	WT-DA (5y), ASK (10y), Thyroid-FA (12y), ERMS-bladder (13y), SLCT-bilateral (15y)	[59]
c.3540C>A **	21	1180	p.Tyr1180Ter	Between PAZ and RNase IIIa	Pathogenic	SLCT (16y), WDFA (16y)	[60]
c.3611_3616delACTACAinsT	21	1204	p.Tyr1204Leu*fs*Ter29	Between PAZ and RNase IIIa	Pathogenic	MNG (26y), SLCT (29y)	[61]
c.3611_3616delACTACAinsT (c.5113G>A)	21 (24)	1204 (1705)	p.Tyr1204Leu*fs*Ter29 (p.Glu1705Lys)	Between PAZ and RNase IIIa (RNase IIIb)	Pathogenic	MNG (26y), SLCT (29y)	[35]
c.3647C>A ***	21	1216	p. Ser1216Ter	Between PAZ and RNase IIIa (RNase IIIb)	Pathogenic	MNG (13y), SLCT (13y)	[62]
c.3726C>A (c.5439G>T)	21 (25)	1242 (1813)	p. Tyr1242Ter (p.Glu1813Asp)	Between PAZ and RNase IIIa (RNase IIIb)	Pathogenic	SLCT (13y), NCMH (21y), PPB (27y), MNG	[50]
c.4050+1delG	Intron 21	-	-	-	Pathogenic	SLCT (9y), MNG (20y), cPNET (20y)	[61]
c.4050+1G>A	Intron 21	-	-	-	Pathogenic	PinB (10y), ERMS-cervix, SLCT, FEP-cervix, FEP-vagina, ERMS-brainstem	[63]
c.4442G>A ^($)^	23	1481	p. Trp1481Ter	Between RNase IIIa and IIIb	Pathogenic	SLCT (6y)	[64]
c.4453A>T (c.5428G>C)	23 (25)	1485 (1810)	p.Lys1485Ter (p.Asp1810His)	Between RNase IIIa and IIIb (RNase IIIb)	Pathogenic	SLCT-bilateral (12y, 14y)	[46]
c.4923_4926delTTTG (c.5438A>G)	23 (25)	1641 (1813)	p. Cys1641Ter (p.Glu1813Gly)	Between RNase IIIa and IIIb (RNase IIIb)	Pathogenic	SLCT (17), MNG, Pineal Cyst, Pituitary Cyst	[42]
c.5018_5021delTCAA (c.5125G>A)	23 (24)	1673 (1709)	p. Ile1673ThrfsTer31 (p.Asp1709Asn)	Between RNase IIIa and IIIb (RNase IIIb)	Pathogenic	SLCT, MNG	[35]
c.5122_5128delGGAGATG	24	1708	p. Gly1708Arg*fs*Ter7	RNase IIIb	Pathogenic	SLCT-bilateral (17y, 27y)	[56]
c.4207-41_5364+1034del (c.5437G>C)	23-24 (25)	1403-1788 (1813)	p. Thr1403_Glu1788del (p.Glu1813Gln)	RNase IIIb (RNase IIIb)	Pathogenic	SLCT (13y), MNG (15y)	[65]
c.5364+1187T>G	Intron 24	1788-1789	p. Glu1788_ L1789insValTer	RNase IIIb	Likely Pathogenic	MNG (12y), SLCT (13y), LC (14y)	[66]
c.5437G>C (c.4626_4626delG) (Left ovarian) (LOH) (Right ovarian)	25 (23)	1813 (1542)	p. Glu1813Gln (p.Gln1542HisfsTer18)	RNase IIIb (Between RNase IIIa and IIIb)	Pathogenic	RC (1.2mo), LC (2.5y),|PinB (7.7y), SLCT-bilateral (13.4y, 15.7y), CBME (17.2y), Nasal-polyps (15.1y), Thyroid-DTC (10.6y)	[31]
c.5441C>T	25	1814	p. Ser1814Leu	RNase IIIb	Pathogenic	Thyroid-Nodule (13y), Thyroid-DTC (18y), SLCT-bilateral (7y, 18y)	[67]
c.5441C>T (c.5125G>A)	25 (24)	1814 (1709)	p. Ser1814Leu (p.Asp1709Asn)	RNase IIIb (RNase IIIb)	Pathogenic	SLCT (11.5y), MNG (12y)	[68]
c.5504_5507delATCC (c.5439G>T)	25 (25)	1835 (1813)	p. Tyr1835SerfsTer2 (p.Glu1813Asp)	RNase IIIb (RNase IIIb)	Pathogenic	cERMS (13y), SLCT (13y), Thyroid-PTC (13y), MNG	[69]
Full gene deletion	all	-	-	all	Pathogenic	SLCT, WT-NOS	[70]
Full gene deletion	all	-	-	all	Pathogenic	cERMS (13y), SLCT (14y), Thyroid-Nodule (13y)	[71]

^&^ Variants are numbered according to the DICER1 cDNA reference sequence (GenBank accession number NM_177438.2), whereby nucleotide +1 corresponds to the A of the ATG-translation initiation codon. ^Δ^ Protein Domains: according to https://www.uniprot.org/uniprot/Q9UPY3. Last access date 23 April 2021. ^◊^ Classification according to VarSome Software (https://varsome.com/. Last access date 23 April 2021). * Both these variants were detected in the same patient. The allelic phase is not known. ** In the same patient, the c.4206+8_4206+ 9insTT variant was identified. The allelic phase is not known. VarSome classifies this variant as “Benign”. The frequency in the general population is ƒ = 0.00964, according to gnomAD Exomes. *** In the same patient, the c. 3649T>A (p.Tyr1217Asn) variant was identified in cis. VarSome classifies this variant as “Likely Benign”. Frequency is not reported in the general population. $ de novo variant. † Patient’s phenotype: MNG: Multinodular Goiter; Polyps-GI: Bowl Polyps; SLTC: Sertoli–Leydig Cell Tumour; cERMS: Embryonal Rhabdomyosarcoma of uterine cervix; PPB type II: Pleuropulmonary Blastoma Type II; Thyroid-PTC: Papillary Thyroid Carcinoma; NCMH: Nasal Chondromesenchymal Hamartoma; Thyroid-DTC: Differentiated Thyroid Carcinoma; PPB: Pleuropulmonary Blastoma, type not specified; JGCT: Juvenile Granulosa Cell Tumour; PPB Type Ir: Pleuropulmonary Blastoma Type I regressed; CN: Cystic Nephroma; PPB type I: Pleuropulmonary Blastoma Type; LC: Lung Cysts; WT-DA: Wilms Tumour, diffuse anaplasia; ASK: Anaplastic Sarcoma of the Kidney; Thyroid-FA: Thyroid Follicular Adenoma; ERMS-bladder: Embryonal Rhabdomyosarcoma of bladder; WDFA: Well-differentiated Fetal Lung Adenocarcinoma; cPNET: Primitive Neuroectodermal Tumour of cervix; PinB: Pineoblastoma; FEP-cervix: Cervical Fibroepithelial Polyp; FEP-vagina: Vaginal Fibroepithelial Polyp; ERMS-brainstem: Embryonal Rhabdomyosarcoma of brainstem. (y): years at diagnosis.

## Data Availability

The data that support the findings of this study are available from open access database and publicly archived datasets. Data are however available from the authors upon reasonable request.
